# Broad spectrum antiviral activity for paramyxoviruses is modulated by biophysical properties of fusion inhibitory peptides

**DOI:** 10.1038/srep43610

**Published:** 2017-03-08

**Authors:** Cyrille Mathieu, Marcelo T. Augusto, Stefan Niewiesk, Branka Horvat, Laura M. Palermo, Giuseppina Sanna, Silvia Madeddu, Devra Huey, Miguel A. R. B. Castanho, Matteo Porotto, Nuno C. Santos, Anne Moscona

**Affiliations:** 1 CIRI, International Center for Infectiology Research, 21 Avenue Tony Garnier, 69365 Lyon Cedex 07, France; 2 INSERM U1111, Lyon, France; 3 CNRS, UMR5308, Lyon, France; 4 Université Lyon 1, Lyon, France; 5 Ecole Normale Supérieure de Lyon, Lyon, France; 6 Instituto de Medicina Molecular, Faculdade de Medicina, Universidade de Lisboa, Av. Prof. Egas Moniz, 1649-028 Lisbon, Portugal; 7 Department of Veterinary Biosciences, College of Veterinary Medicine, Ohio State University, Columbus, USA; 8 Department of Pediatrics, Columbia University Medical Center, 701 W. 168th St., New York, NY, USA; 9 Center for Host-Pathogen Interaction, Columbia University Medical Center, 701 W. 168th St., New York, NY, USA; 10 Department of Biomedical Sciences, University of Cagliari, Cittadella Universitaria, Monserrato, Cagliari, Italy; 11 Department of Microbiology & Immunology, Columbia University Medical Center, 701 W. 168th St., New York, NY, USA; 12 Department of Physiology & Biophysics, Columbia University Medical Center, 701 W. 168th St., New York, NY, USA.

## Abstract

Human paramyxoviruses include global causes of lower respiratory disease like the parainfluenza viruses, as well as agents of lethal encephalitis like Nipah virus. Infection is initiated by viral glycoprotein-mediated fusion between viral and host cell membranes. Paramyxovirus viral fusion proteins (F) insert into the target cell membrane, and form a transient intermediate that pulls the viral and cell membranes together as two heptad-repeat regions refold to form a six-helix bundle structure that can be specifically targeted by fusion-inhibitory peptides. Antiviral potency can be improved by sequence modification and lipid conjugation, and by adding linkers between the protein and lipid components. We exploit the uniquely broad spectrum antiviral activity of a parainfluenza F-derived peptide sequence that inhibits both parainfluenza and Nipah viruses, to investigate the influence of peptide orientation and intervening linker length on the peptides’ interaction with transitional states of F, solubility, membrane insertion kinetics, and protease sensitivity. We assessed the impact of these features on biodistribution and antiviral efficacy *in vitro* and *in vivo.* The engineering approach based on biophysical parameters resulted in a peptide that is a highly effective inhibitor of both paramyxoviruses and a set of criteria to be used for engineering broad spectrum antivirals for emerging paramyxoviruses.

Human parainfluenza viruses (HPIVs) are paramyxoviruses belonging to the respirovirus (HPIV1 and 3) or rubulavirus (HPIV2 and 4) genera that cause human respiratory diseases including bronchitis, bronchiolitis, and pneumonia in infants, children, or immune-compromised individuals. The HPIV species viruses are responsible for 30–40% of all acute respiratory tract infections in infants and children^1^. Nipah virus (NiV) is a zoonotic paramyxovirus of the henipavirus genus that represents a global health risk with broad, unpredictable pandemic potential. Infection with this virus is devastating, rapidly causing a generalized vasculitis leading to lethal encephalitis and serious respiratory infections. The common natural reservoirs of henipavirus genus members (including NiV, Hendra virus, Cedar virus and numerous newly identified species) are fruit bats^2^,^3^, but recent identification of rodent-origin henipa-like viruses that cause lethal respiratory disease in humans suggests that the range of natural mammalian hosts may be even broader^4^. Transmitted by air or food, its mechanism of infection is complex, and no drugs exist to prevent or treat infection. The recent human-to-human spread of NiV in Asia has raised the real possibility of global spread. NiV and HPIV, while different in their associated diseases and geography, infect using similar mechanisms and are vulnerable to the same antiviral strategy.

Paramyxovirus infections are initiated by fusion between viral and host cell membranes. For HPIV3, NiV, and other paramyxoviruses, the surface glycoproteins play critical roles in the initial events of viral infection, mediating virion attachment to cells and fusion of the viral and cellular membranes. The viral fusion protein (F), upon insertion of the fusion peptide – formed after cleavage of the precursor F into its final fragment – into the host target cell membrane, forms a transient intermediate to pull the viral and cell membranes together. Two heptad-repeat regions that are present in each ectodomain of F refold to form a highly stable, antiparallel six-helix bundle structure that is coupled to membrane fusion. The structure required for this step can be specifically targeted by fusion-inhibitory peptides. The N-terminal repeat (HRN) is adjacent to the hydrophobic fusion peptide that inserts into the target cell membrane during the fusion process. The C-terminal repeat (HRC) immediately precedes the transmembrane domain. A folding-back of the F ectodomain drives the viral membrane towards the cell-associated fusion peptide to bring the two membranes into proximity, causing membrane fusion and viral entry.

Peptides derived from the HRC of paramyxovirus F proteins interfere with formation of the six-helix bundle in a dominant-negative manner by binding to the transiently exposed HRN coiled coil in the transient fusion intermediate, thereby inhibiting membrane fusion^5^. We have shown the potential of modified fusion inhibitor peptides as NiV and HPIV antiviral agents, and showed that a sequence corresponding to the HRC domain of HPIV3 effectively inhibits both HPIV3 and NiV^6^. Interhelical packing interactions in the post-fusion core of the NiV and HPIV3 F proteins are important determinants of viral entry and its inhibition. Fusion inhibitor peptides can be engineered at the level of these parameters, for optimal inhibition, increasing potency for both viruses^6^,^7^,^8^,^9^,^10^. Conjugating cholesterol to a fusion inhibitor peptide enhances NiV antiviral activity up to 100-fold, by targeting the peptide to the plasma membrane where fusion occurs^8^ and endows the fusion inhibitor with the ability to localize to the central nervous system, successfully preventing and treating NiV infection in a golden hamster model of disease^6^. Surprisingly, the most effective antiviral peptides for NiV and the related Hendra virus (HeV) are those derived from the F of HPIV3. The antiviral strategy discussed here exploits the uniquely broad spectrum antiviral activity of these HPIV3 derived inhibitory peptides to treat paramyxovirus infection. In an effort to understand the biophysical properties that correlate with peptide efficacy and thereby improve upon their antiviral utility, we explored biophysical features of the lipid-conjugated peptides that alter aggregation and membrane insertion kinetics. We have identified several properties that correlate with the peptides’ potency and permit enhancement of antiviral effect for both HPIV3 and NiV.

## Results

**Figure 1:**
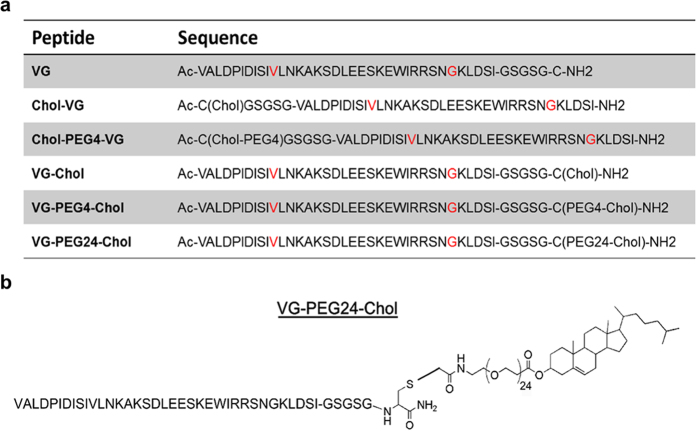
Sequences and structures of the HPIV3 HRC peptides. (**a**) The peptides consist of the HPIV3 HRC (amino acids VALDPIDISIVLNKAKSDLEESKEWIRRSNGKLDSI-GSGSG-C of HPIV3 F with a GSGSG spacer and C for thioether reaction). Cholesterol was conjugated to the peptides at either the N- or C- terminus either with or without a PEG linker. Residues in red were modified from the original F-protein derived peptide sequence. (**b**) Schematic representation of the HPIV3 HRC with cholesterol conjugated at the C-terminus and a linker of 24 PEG moieties “VG-PEG24-Chol”.

We previously showed that HPIV3 HRC-derived sequences interact with HPIV3 F and NiV F HRN, that antiviral potency can be improved by sequence engineering and cholesterol conjugation, and that peptides without linkers between the protein and lipid component are less potent than those with a 4 polyethylene glycol (PEG) linker in the intervening region^6^,^8^. Here we investigated the impact of the peptide orientation, as well as the influence of the PEG linker length, on the peptide antiviral efficacy. We focus on PEG linkers for exploration of these mechanisms since PEG features a number of characteristics that may be critical for tissue penetration of cholesterol-tagged peptides. PEG is soluble in both aqueous and organic media, and thus compatible with both plasma/extracellular fluids and membrane environment. Unlike alkyl linkers, PEG linkers do not cause aggregation and precipitation in the compounds in which they are incorporated. PEG is used to prevent non-specific adsorption onto hydrophobic surfaces, being non-toxic and non-immunogenic. The amino acid sequence and the modifications introduced into the peptides used for this study are shown in Fig. 1a. The entire set of peptides used here is based on a single peptide with the “VG” sequence, which has E549V^9^ and Q479G introduced into our published HPIV3 F HRC-derived peptide^10^. As for the previously reported NiV, HPIV3 and HIV inhibitors^10^, reaction of the core sequence with a bromoacetyl derivative of cholesterol featuring a 4, 12 or 24-unit PEG spacer (PEG_4, 12 or 24_) or without linker produced the cholesterol-conjugated monomer. The peptide with the “VG” sequence and a 24-unit PEG spacer before the cholesterol (called VG-PEG24-Chol) is shown in Fig. 1b.

### Impact of PEG linker length and peptide orientation on HPIV3 and NiV cell-cell fusion

**Figure 2:**
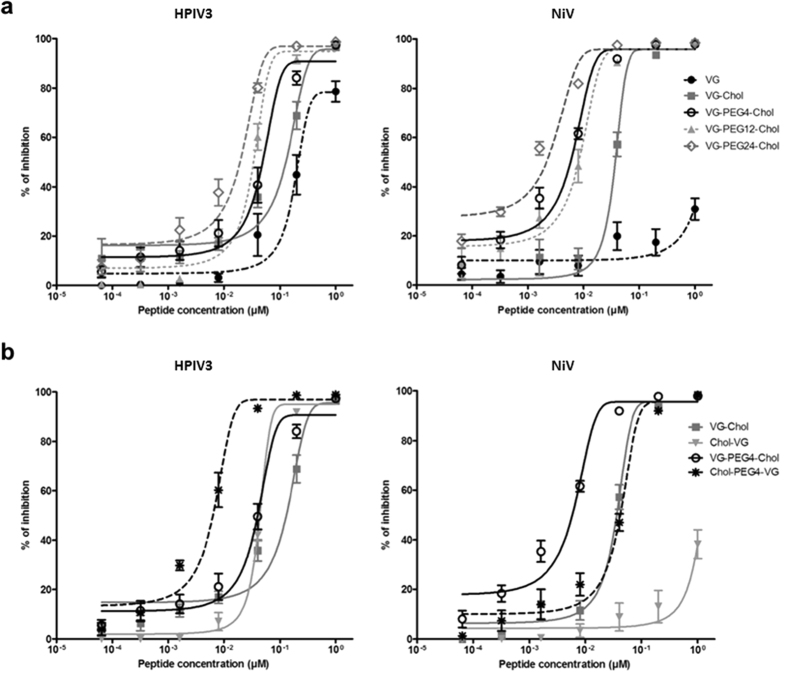
Influence of spacer length and sequence orientation on the inhibition of HPIV3 HN/F and NiV G/F mediated fusion by HPIV3 F derived HRC peptides. Fusion of HPIV3 HN/F (panel a left) or NiV G/F-coexpressing cells (panel a right) with 293T cells in the presence of serial dilutions of VG, VG-Chol, VG-PEG4-Chol, VG-PEG24-Chol was quantified at 1 h, using a β-galactosidase complementation assay. (**b**) The same assay was used to quantify fusion in the presence of N-terminally versus C-terminally conjugated peptides. Results are presented as percent reduction in luminescence (y-axis) compared with no treatment. Each point is the mean ± s.d. of results with *n* = 3 experiments.

VG sequence peptides, either unconjugated or conjugated with cholesterol at the C-terminus, with linker lengths ranging from 0 to 24 PEG, were assessed for their ability to inhibit the cell-cell fusion mediated by viral glycoproteins. A quantitative cell-cell fusion assay based on β-galactosidase (β-gal) complementation was used as previously described^11^. Cholesterol conjugation improved peptide inhibition of cell-cell fusion mediated by HPIV3 and NiV glycoproteins, as expected. Incorporation of a PEG_4_ spacer significantly enhanced peptide efficacy against both viral glycoproteins. Increasing the linker length to PEG_12_ did not improve upon this result. The peptide with the longest spacer tested – PEG_24_ –showed the highest potency in this assay for both NiV and HPiV3 glycoproteins (Fig. 2a) suggesting that the relationship between spacer size and efficacy at inhibiting fusion is not linear.

The impact of the orientation of the peptide’s sequence on its ability to block cell-cell fusion was explored (Fig. 2b) by conjugating the lipid moiety and PEG spacer to either the C-terminus (e.g., VG-Chol) or the N-terminus (e.g., Chol-VG). All conjugated peptides showed potency against HPIV3 in this assay, with the N-terminally conjugated peptides significantly more effective at blocking fusion. For NiV, in contrast, Chol-VG has too little effect to determine an IC50 against NiV fusion. Only C-terminally conjugated peptides blocked the NiV glycoprotein-mediated fusion with high efficacy in the presence of the PEG linkers. The efficacy of the HPIV3 HRC-derived peptide against HPIV3 itself is higher when spacer and lipid are conjugated N-terminally, but this results in a loss of multipotency against NiV. These results suggest that both orientation of the peptide sequence and the length of the spacer are important for antiviral multipotency.

### HPIV3 F HRC-derived peptides interact with uncleaved HPIV3 F

**Figure 3:**
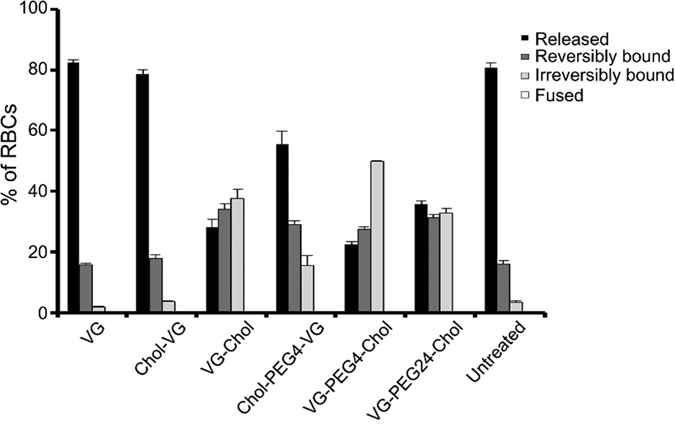
Spacer and peptide orientation influence on the ability of HPIV3 F HRC cholesterol-tagged peptides to capture fusion intermediates of native F before fusion peptide insertion in the target membrane. When HN is receptor bound, unprocessed F (K108G; cleavage site mutant csm) may be activated to a transient intermediate that is captured by cholesterol-conjugated peptides. Monolayers of cells coexpressing HN and unprocessed F (csm) were interacted with receptor-bearing RBCs at 4 °C. Upon transfer to 37 °C, medium containing either no peptide or 1 μM peptide was added. Zanamivir was added to disengage HN from its receptor after 1 h, and the values on the *y* axis reflect the quantitation of RBCs that were directly released (black), reversibly bound by HN-receptor interaction (gray), irreversibly bound (light gray), or fused (white). The values are means (±standard deviations) of results from three experiments.

In previous studies, we used HRC-derived lipid-conjugated peptides to investigate the conformational flexibility of uncleaved F protein (F in its precursor protein form, with the hydrophobic fusion domain still buried)^12^. The peptides bind to their corresponding HRN domains only when they are exposed by the unfolding of F, and serve as a tool to identify this exposure. For these experiments, peptides are attached to red blood cell (RBC) membranes via their cholesterol tails, and the RBCs are allowed to interact with cells expressing an uncleaved mutant of HPIV3 F (K108G) along with HN to engage the sialic acid receptor on the RBCs and initiate F-activation. The read-out for peptide interaction with the F protein’s HRN is adherence of the RBCs to the F-expressing cells. Since the uncleaved F cannot insert into the RBC membrane, this adherence occurs only via a peptide “bridge” formed when the peptide that is anchored to the RBC via its lipid tail also interacts with the HRN sequence of F expressed on the transfected cells^12^. The uncleaved F has enough flexibility to unfold and expose the HRN domain upon activation by HN, but cannot fully extend into the transient “pre-hairpin” intermediate with its hydrophobic end inserted into the target membrane^12^. For exploring peptide function, this assay provides a measure of the efficiency with which peptides bind at an early stage in the fusion process before the F-insertion step. Here we used the “peptide bridging” assay to assess the interaction of the series of modified VG peptides with the early transitional steps of F protein (Fig. 3). 293T cells co-expressing HPIV3 HN and uncleaved F (cleavage site mutant K108G, csm) were allowed to interact with receptor-bearing RBCs at 4 °C, then transferred to 37 °C in the presence of either C-terminally or N-terminally conjugated peptides (1 μM) with or without PEG_4_ linkers. Zanamivir, a sialic acid analog that blocks HN-receptor interaction^13^,^14^ was added after 1 h to disengage cells that were bound by HN-receptor engagement alone. The proportion of RBCs that were either free, reversibly bound by HN-receptor interaction (and therefore released by zanamivir), irreversibly bound by peptide bridging, or fused was quantified.

The unconjugated VG and the N-terminally conjugated Chol-VG peptides both failed to bind the HRC of uncleaved F in a manner sufficient to “bridge” the F-expressing cells and receptor-bearing cells; 80% of the RBCs were free and 20% were reversibly bound (attached only by HN-receptor engagement) in their presence. In contrast, VG-Chol (C-terminally lipid conjugated) peptide bridged the cell populations by attaching the F protein to the plasma membrane of the RBCs, as suggested by the 35% of irreversibly bound RBCs in the presence of this peptide. PEG spacers in the peptides led to an increase in bridging (as seen by irreversible RBC binding), for both the C-terminally and the N-terminally conjugated peptides, with the C-terminally conjugated peptides being significantly more effective at interacting with the early transitional intermediate of F. Since the spacer-less Chol-VG failed to bridge, this result suggests that the spacer permits the N-terminally conjugated peptide to insert into the RBC membrane. Of the peptides tested, VG-PEG4-Chol showed the most effective bridging, more than 50% irreversible retention, while the peptide that was most effective at inhibiting fusion inhibition, VG-PEG24-Chol (Fig. 2), looks similar to VG-Chol, a far less effective inhibitor. As further explored below, this may relate to the more dynamic membrane insertion of VG-PEG24-Chol, allowing release of the “bridge” mediated by this peptide and release of attached RBCs.

### PEG linkers improve the solubility of HPIV3 derived peptides

**Figure 4:**
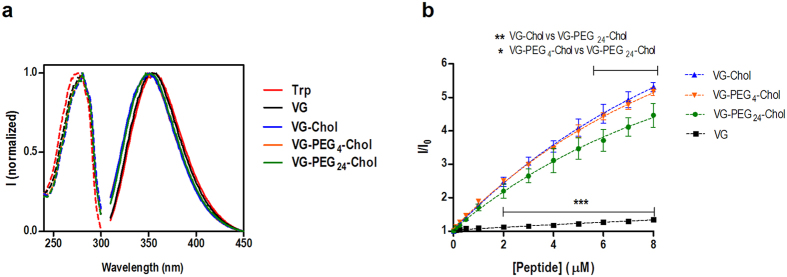
Spectroscopic characterization of VG peptides. (**a**) Environmental effects on the tryptophan residue of VG peptides. Normalized fluorescence excitation (dashed line) and emission (solid line) spectra of each peptide and Trp in HEPES buffer 10 mM pH 7.4 in NaCl 150 mM. (**b**) Concentration-dependent aggregation of the VG peptides, evaluated by ANS (12.8 μM) fluorescence intensity (λexc = 369 nm; emission spectra integrated from 400 to 600 nm). The values are means ± standart error of the mean (±s.e.m) of at least three experiments. Statistical analysis was performed using a 2-way ANOVA test (*P < 0.05; **P < 0.01; ***P < 0.001).

The aggregation and lipid-binding properties of VG peptides were assessed based on the intrinsic fluorescence of their tryptophan (Trp) residues (Fig. 4a). The fluorescence excitation spectra of VG peptides in buffer nearly overlap that of free Trp. Regarding the fluorescence emission spectra, only the untagged peptide VG showed a close overlap with Trp spectrum. Cholesterol-containing VG peptides presented a blue-shift on the emission maximum, indicating that the Trp residue of these conjugates are in a more hydrophobic environment, as previously observed for different cholesterol-tagged HIV fusion inhibitor peptides^15^,^16^.

Fusion inhibitor aggregation propensity was tested using the fluorescent dye 1-anilino-8-naphthalene sulfonate (ANS). In the presence of hydrophobic pockets, there is a significant increase in the ANS quantum yield and a blue-shift in the maximum emission peak is observed. As expected, the untagged peptide VG was unable to form aggregates (Fig. 4b). Titration of ANS with VG-Chol, VG-PEG4-Chol and VG-PEG24-Chol at pH7.4 led to a significant blue-shift of 67 nm, 63 nm and 58 nm, respectively (data not shown). This is indicative that all cholesterol-tagged peptides form aggregates when dissolved in HEPES 10 mM pH7.4 NaCl 150 mM buffer. VG-Chol, VG-PEG4-Chol and VG-PEG24-Chol were compared in order to test the PEG spacer’ effect on peptide aggregation (Fig. 4b). The longer the PEG spacer the lower the aggregation observed. This variation is more evident upon comparing VG-Chol with VG-PEG24-Chol (Fig. 4b).

### PEG linkers enhance HRC-derived peptides’ insertion in lipid membranes

**Figure 5:**
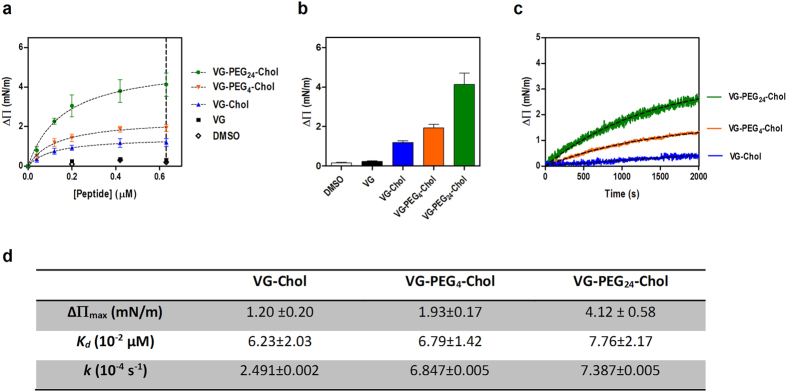
Lipid monolayers surface pressure perturbation. (**a**) Changes in surface pressure as a function of VG peptides concentration or DMSO addition to pure POPC monolayers. (**b**) Changes in surface pressure at 0.63 μM of each VG compound or DMSO on POPC. (**c**) Variation of the surface pressure of POPC monolayer as a function of time after the injection of VG peptides at a final concentration of 0.2 μM. Table below figure shows ΔΠ_max_, dissociation constants, *K*_*d*_, and kinetic adsorption rate constants, *k.*

To quantify peptide-membrane affinity and assess the changes induced by cholesterol conjugation, we evaluated the ability of VG series peptides to induce changes on the surface pressure of lipid monolayers (Fig. 5). As shown in Fig. 5a, VG peptides are able to induce changes on the surface pressure of 1-palmitoyl-2-oleoyl-*sn*-glycero-3-phosphocholine (POPC) monolayers. Only VG and the peptide solvent control with dimethyl sulfoxide (DMSO) showed no changes on the lipid monolayer. VG-PEG24-Chol was the most active peptide by inducing the largest change on the monolayer surface pressure, with ΔΠ_max_ ~ 4 mN/m (Fig. 5b). Increasing concentrations of VG peptides led to an increase on the surface pressure, until a plateau of peptide saturation on the monolayer is reached. These data allow the use of equation (1) to calculate the dissociation constant, *K*_d_. Despite the similar affinity for POPC (Fig. 5d), VG peptides presented very distinct kinetics towards this lipid (Fig. 5c). Using equation (2), a higher insertion rate, *k*, was observed for VG-PEG24-Chol (7.4 × 10^−4^ s^−1^), in comparison with VG-PEG4-Chol and VG-Chol (Fig. 5c,d). A similar kinetic behavior was observed for a mixture of POPC with 33% of cholesterol, which represents more ordered domains and the membranes of some cells with high cholesterol content, such as RBCs (data not shown).

**Figure 6:**
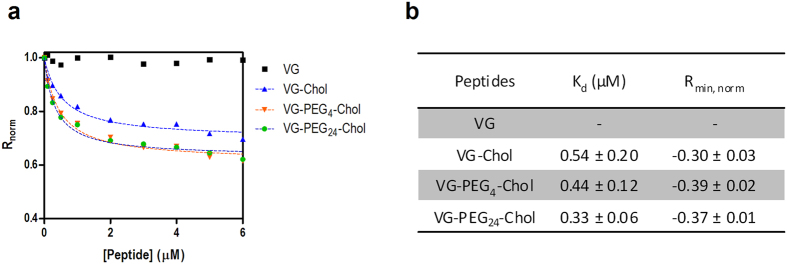
Peptide affinity towards RBCs assessed by di-8-ANEPPS fluorescence. (**a**) The dependence of the ratio *R* on VG (black), VG-Chol (blue), VG-PEG4-Chol (orange) and VGPEG24-Chol (green) concentrations for RBCs was analyzed by a single binding site model (dashed lines) in order to quantify the apparent dissociation constants, *Kd.* The *R* values were normalized for the initial value of zero peptide concentration. (**b**) The *Kd* values represent the mean ± s.e.m. (*n* = 4). Rmin is the asymptotic minimum value of *R*.

RBCs were selected as cell model to test peptide-cell membrane affinity. However, a direct measurement of the peptide tryptophan fluorescence is impracticable with cells. As an indirect reporter, we used the lipophilic fluorescent probe di-8-ANEPPS to measure the interaction of the peptides with the cell membranes. RBCs were labeled with this probe as previously reported^15^,^16^,^17^. The VG peptides were incubated with the RBCs during 1 h. The cholesterol-tagged peptides were able to induce changes on the membrane dipole potential sensed by di-8-ANEPPS (Fig. 6). The VG peptide and the control DMSO did not cause any change on the membrane dipole potential. As a quantitative measure of the interaction of VG peptides with membranes, the ratio *R* was measured for a range of peptide concentrations. This ratio decreases upon increasing the concentration of cholesterol-tagged peptides, following a hyperbolic curve. These curves were fitted with equation (3), allowing determination of the dissociation constant, *K*_*d*_. VG-PEG24-Chol showed a higher affinity (*K*_*d*_ = 0.33 μM) in comparison with VG-Chol (*K_d_* = 0.54 μM) and VG-PEG4-Chol (*K*_*d*_ = 0.44 μM) (Fig. 6B).

### Protease sensitivity of HPIV3 derived peptides

**Figure 7:**
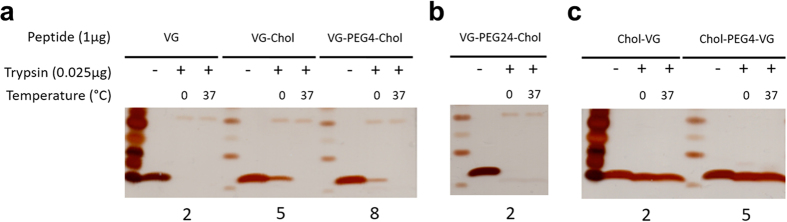
Protease sensitivity of HPIV3 derived peptides. The indicated peptides were incubated in the absence (−) or presence (+) of trypsin, at 0.025 µg/µl for 1 hr at 0 °C or 37 °C. The products of the reaction were subjected to reducing SDS-page gels and silver stained.

We recently tested the hypothesis that while lipid conjugation decreases protease sensitivity, addition of PEG linkers increases protease sensitivity. Figure 7 shows VG series peptides incubated in the absence (−) or presence (+) of trypsin, at 0.025 µg/µl for 1 hr at 0 °C or 37 °C. Cholesterol conjugation to VG decreased protease degradation at 0° (compare panel A lane 2 to lanes 5 and 8). Adding the PEG24 linker and cholesterol at the C-terminus increased degradation significantly (panel B lane 5). However when the cholesterol (with or without PEG linker) is conjugated to the N-terminus, the peptides were significantly more resistant to protease degradation. Panel C shows that even at 37° the peptides with N-terminal tags are not degraded.

### Impact of PEG spacer length on the inhibition of HPIV3 and NiV infection plaque formation in monolayer cultured cells

**Figure 8:**
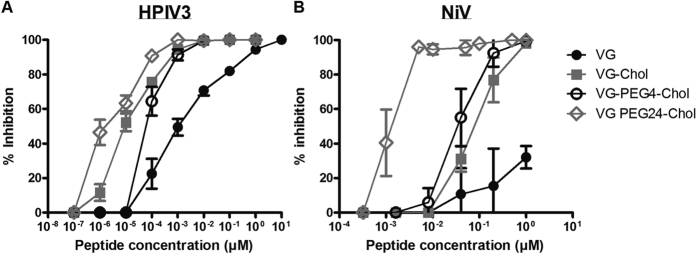
Spacer length impacts VG peptide efficacy against both HPIV3 clinical isolates (CI) and NiV. (**a**) Vero cell monolayers were infected with HPIV3 at a multiplicity of infection (m.o.i.) of 2.5 × 10^−3^, in the presence of increasing concentrations of the peptide. After a 90-min incubation at 37 °C, cells were overlaid with methylcellulose, and plaques were immunostained and counted after 24 h. The percent inhibition of viral entry (compared to results for control cells infected in the absence of inhibitors) is shown as a function of the (log-scale) concentration of peptide (*n* = 3 separate experiments). (**b**) Vero cell monolayers were infected with wild type NiV at an m.o.i. of 2.5 × 10^−3^ in the presence of increasing concentrations of peptides. After 1 h incubation at 37 °C, cells were overlaid with methylcellulose, and plaques were stained and counted after 96 h. The percent inhibition of viral entry (compared to results for control cells infected in the absence of inhibitors) is shown as a function of the (log-scale) concentration of peptide. Data points are means ± s.d. (*n* = 6 separate experiments).

To explore the significance of these observations in the context of authentic virus, we assessed the efficacy of VG series peptides at inhibiting infection by HPIV3 and live NiV, in cell culture (Fig. 8). As expected, cholesterol conjugation significantly ameliorated the peptide efficacy against both viruses, as measured by reduction in plaque formation. While the presence of the PEG4 linker had no impact on the peptide’s IC90 against HPIV3 (~1 nM) it reduced the IC90 for inhibition of NiV (from 300 nM to 50 nM). An increase in spacer length to PEG24 led to a significant increase of efficacy against both HPIV3 (IC90 ~ 0.1 nM) and NiV (IC90 ~ 2 nM). In addition, we evaluated the toxicity and specificity of the peptides. Table 1 shows that toxicity of VG-PEG24-Chol on cells appears only when we reach a concentration of 1000 nM (10,000 × The IC90); toxicity of the other peptides in the series was even lower. Since VG-PEG24-Chol, although quite non-toxic, had the highest toxicity of all the peptides in this series, we chose this peptide for an experiment designed to assess specificity of inhibition. We compared the antiviral effect of VG-PEG24-Chol to PEG24-Chol conjugated peptides that are effective against measles, influenza virus, and feline immunodeficiency virus. None of these peptides were inhibitory for HPIV3, supporting the notion that potency depends on the presence of the HPIV3 peptide sequence, not simply on the linker or lipid moiety (Table 1).

### Impact of PEG spacer length on the inhibition of HPIV3 in HAE

HAE cultures are an authentic model of human lung that can be used to evaluate features of infection and antiviral efficacy. Peptide efficacy in HAE seems to correlate with *in vivo* efficacy^6^,^18^. Therefore we now assessed efficacy of VG peptides in HAE, using this authentic lung model to compare those peptides that have the best potency in monolayer culture and no toxicity at a 100 μM concentration in HAE cultures. VG-PEG4-Chol, Chol-PEG4-VG, and VG-PEG24-Chol were compared for their ability to inhibit infection in HAE at 10 μM. A single dose of 10 μM of VG-PEG4-Chol decreased the viral titer of HPIV3 by approximately two logs at day 2 after infection and approximately one log at day 5. For Chol-PEG4-VG and VG-PEG24-Chol, no virus was produced at any time after infection, indicating that viral infection was completely inhibited at this concentration in HAE. When the inhibitors were given in a single dose of 1 μM, VG-PEG24-Chol resulted in complete inhibition of infection; however, both Chol-PEG4-VG and VG-PEG4-Chol showed only partial inhibition (data not shown).

### Peptide biodistribution *in vivo*

**Figure 9:**
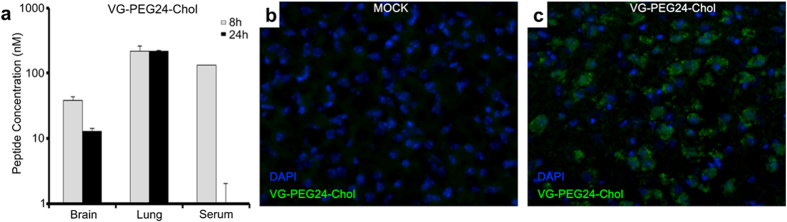
Biodistribution of cholesterol-tagged VG-PEG24-Chol peptide. Golden hamsters were injected with 2 mg/kg of VG-PEG24-Chol peptide intraperitoneally (i.p.). (**a**) At indicated times (x-axis) the animals (*n* = 3/data point) were sacrificed, and the peptide concentrations in plasma, lung, and brain were quantitated. The ordinate values are means (±s.d.) of results from three animals. (**b**,**c**) Immunofluorescent staining of VG-PEG24-Chol (in green) in the brain of hamsters 24 h after i.p. administration. Nuclei were counterstained using DAPI. Peptide staining is absent from untreated animals (**b**). VG-PEG24-Chol peptide (green) is present in the brain parenchyma of treated hamsters (**c**), confirming its ability to reach the CNS.

The utility of peptides as antivirals depends in large part on biodistribution and availability at the site of infection. We have previously assessed the biodistribution of the HPIV3 derived peptides in hamsters and cotton rats that serve as the model for NiV and HPIV3 infection^6^,^19^. Here, we assessed the half-life and distribution of the VG-PEG24-Chol peptide (2 mg/kg, i.p.) and detected free peptide in the serum at 8 h, with concentration peaking at 120 nM before dropping after 24 h, accompanied by peptide detection at 24 h in organs (Fig. 9a). No peptide was detected in the urine, suggesting the peptide is not eliminated intact (data not shown). Immuno-histofluorescence showed that peptide is present in brain parenchyma 24 h after administration, confirming its ability to reach the CNS (Fig. 9b and c). We had previously observed CNS localization not only for the HPIV3 peptide^6^ but also for measles virus (MV) F sequence derived peptides^11^, suggesting that this feature does not require a highly specific peptide sequence.

### Peptide inhibition of HPIV3 and NiV infection *in vivo*

**Figure 10:**
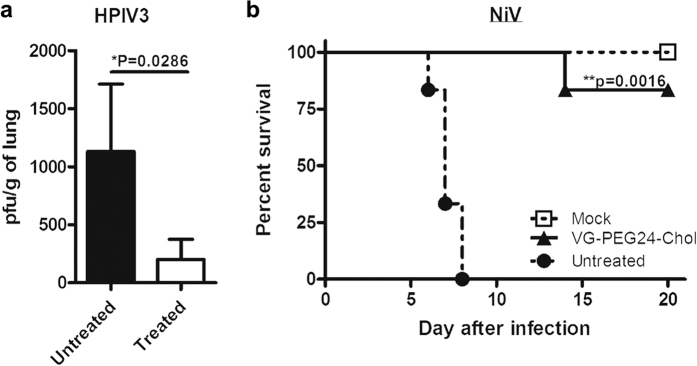
*In vivo* efficacy of cholesterol-tagged VG-PEG24-Chol peptide. (**a**) Cotton rats (*n* = 4) received 2 s.q. injections of peptide/day from day −1 to day 2 post intranasal infection with HPIV3 (10^6^ pfu/animal). Animals were euthanized after 3 days and virus titers were determined from lung homogenates in plaque assays. Peptide significantly reduced viral load (*p = 0.0286, using Mann-Whitney U-Test). (**b**) Peptide i.p. injections were given to groups of 6 hamsters from day −1 to day 10, concurrently with 100LD50 of live NiV (day 0). Untreated animals were injected with vehicle alone, while mock did not receive injection. Animals were followed for 3 weeks. Peptide significantly improved survival (**P = 0.0016, using Mantel-Cox test).

Having established peptide biodistribution in cotton rats and hamsters, we assessed VG-PEG24-Chol efficacy against HPIV3 and NiV infection in the corresponding models. Peptide administered s.q. significantly decreased the viral load in cotton rat lungs 3 days after intranasal infection with HPIV3 (p = 0.0286, using Mann-Whitney U-Test; Fig. 10a). For NiV, i.p. VG-PEG24-Chol peptide administration daily from day-1 to day 10 after NiV inoculation (100LD50, i.p.) significantly improved the survival of hamsters given a lethal infection inoculum (p = 0.0016, using Mantel-Cox test; Fig. 10b). These results suggest that the peptide identified as bearing optimal biophysical properties for *in vitro* inhibition is promising as an inhibitor of infection and is effective against both viruses *in vivo*.

## Discussion

We have shown that specific, predictable features are important for paramyxovirus antiviral peptide efficacy, and we have now begun to pinpoint the contributions to efficacy of amino acid sequence and lipid moiety, as well as linker length, solubility and other biophysical features. We build upon the remarkable foundation of a peptide sequence derived from the HRC domain of one paramyxovirus, HPIV3, which inhibits both HPIV3 and the quite different paramyxovirus, NiV.

The fluorescence spectra of the VG series peptides (Fig. 4) showed that the conjugation of cholesterol leads to a change on the microenvironment of the fluorescent tryptophan residues. These results indicate a more hydrophobic setting surrounding the tryptophan, suggesting a possible peptide aggregation. The data from the ANS experiments (Fig. 4) confirmed this hypothesis, showing that incorporating PEG24 on the VG series peptides partially decreases aggregation. In a recent work on PEGylation, it was reported that the conjugation of PEG to Aβ peptides enhanced peptide solubility and led to concentration-dependent reversible fibrillization^20^.

A longer PEG linker promoted a faster kinetic towards lipid monolayers as observed in Fig. 5. In addition to the presence of cholesterol, the length of the linker was also a key factor contributing to the membrane driving force. In agreement with previous results, a less aggregated peptide could diffuse more easily towards the cell membrane, which is beneficial for peptide local concentration.

A high affinity for RBCs membrane was observed for VG-PEG24-Chol (Fig. 6), corroborating the lipid monolayers and the aggregation experiments. We have previously shown that cholesterol conjugation to HIV-1 fusion inhibitor peptides also promotes the interaction with RBCs^15^,^16^. Peptides lacking membrane binding domains are optimal candidates for cholesterol tagging, as their antiviral activity is boosted after that. The inclusion of PEG spacers seems to reduce peptide aggregation and induce a fast kinetics towards cell membranes.

When we correlate these biophysical analyses with antiviral activity, data suggest that a synergic combined effect of the membranotropic behavior provided by the cholesterol addition and the increase in solubility (decreased tendency to aggregate) conferred by PEG addition explains the antiviral pattern found for these compounds. The optimal peptide must have the correct balance between membrane tropism and solubility.

The most effective peptides we tested for HPIV3 and NiV are those with the fastest kinetics of insertion and release from membranes, a feature that correlates with the PEG linker length. However, a long PEG linker is expected to cause the peptides to be more exposed to the aqueous environment^15^,^16^, and more sensitive to protease degradation, as shown in Fig. 7, and thereby to have a shorter half-life. In other words, it seems that since the pocket binding domain of the antiviral peptide is more exposed it is more potent, but also more sensitive to protease. This could potentially explain why the level of VG-PEG24-Chol in the brain and serum is relatively low compared to our previous data for other peptides^6^,^19^. The finding that the longer PEG increases protease sensitivity is part of the complexity of identifying ideal candidates for *in vivo* testing; one must identify a balance between absolute antiviral potency and stability. In order to be able to take advantage of the antiviral potency conferred by the longer linker (PEG24) we are now addressing ways to reduce protease sensitivity, for example by modifying the protein backbone.

Surprisingly, when the cholesterol (with or without PEG linker) is conjugated to the N-terminus, the peptides are significantly more resistant to protease degradation (Fig. 7). Lipid conjugation is known to enhance the helicity of peptides^21^, and we speculate that the differential effect of conjugating at the N- and C-termini may relate to the fact that the N-terminus of this peptide is less structured than the C-terminus^6^,^22^, and therefore more likely to be protease sensitive. Conjugation at this less-structured terminus may thereby be protective either due to the effects of aggregation or of enhanced helicity.

**Figure 11:**
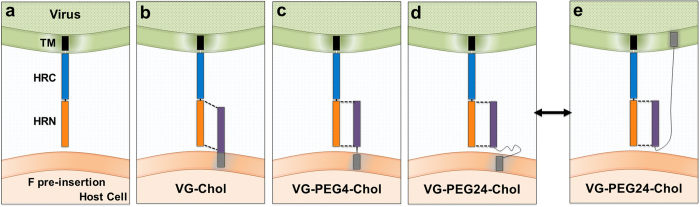
Schematic diagram of implications of PEG spacer effect on lipid-conjugated peptides’ interaction with pre-insertion F. The panels show the interaction of each cholesterol-tagged peptide with the uncleaved HPIV3 F molecule. (**a**) The uncleaved F protein does not insert into the target membrane. (**b**) VG-Chol without PEG linker is misaligned with the HRN domain of F. (**c**), (**d**) PEG linkers permit alignment with the HRN domain of F before insertion, and the longer linker may permit HRN interaction when inserted in cis on the viral membrane (**e**).

While until now we have focused the antiviral strategy on the peptides with lipid conjugated to the C-terminus because they were far more potent inhibitors compared to those with lipid conjugation at the N-terminus^8^, here we showed that by adding a PEG linker between the lipid and the peptide sequence at the N-terminus, the N-terminally conjugated peptides are rendered more potent against the virus from which they are derived. The efficacy of N-terminally lipid conjugated fusion inhibitory peptides has been studied for both Ebola^21^and Newcastle Disease virus^23^. In the case of Newcastle Disease virus, Li *et al*. showed that N-terminal cholesterol conjugation enhanced the potency of inhibitory peptides and allowed them to penetrate the CNS, while the effect of addition of a PEG3 linker between the N-terminal cholesterol and the inhibitory peptide was less clear. In our case, the linker between N-terminal cholesterol and peptide significantly enhances anti-HPIV3 potency, while abrogating broad-spectrum antiviral activity. We speculate that without a flexible linker, the N-terminally conjugated peptides could not attain the proper position for interacting with the target region on F - or could do so only when not anchored in the membrane or later in the fusion process (after fusion peptide insertion and during the refolding step). The mechanistic basis for the negative effect on inhibition of NiV remains to be explored. As shown schematically in Fig. 11, a flexible PEG linker allows for freedom of orientation in order to align properly with the target HRN domain, or for more dynamic kinetic of insertion into the membrane. This may be an advantage not only for interacting with the F protein once it has inserted into the host cell membrane (at which point it is a target for both lipid-conjugated and unconjugated peptide inhibitors)^6^ but also for interacting with the pre-insertion F. The pre-insertion F protein is shown in Fig. 11 as extended into a transient intermediate in panel a; panels b, c, and d depict the C-terminally conjugated VG-Chol peptides with each linker length interacting with this pre-insertion step in fusion, suggesting that the longer linker may allow for optimal alignment between the peptide and the HRN on F. The peptide with a PEG-24 linker (Fig. 11d,e) has been shown to be the most effective inhibitor but to less potently capture early intermediates of F activation (see Fig. 3). Based on the biophysical data showing the increased dynamic membrane interaction for this peptide, we speculate that this peptide may flip between the target host cell (panel d) and the viral membrane (panel e), reducing observed capture of F while increasing efficacy. These mechanisms could also explain why the N-terminally conjugated peptides may be rendered effective by a long linker which could either optimize alignment with F or permit activity from both cell and viral surfaces. In this study, we focused on peptides with the lipid conjugation at the C-terminus because this approach led to conservation of the broad antiviral spectrum – a primary goal of this work. However in light of the advantage shown by the N-terminally conjugated peptides in terms of protease sensitivity, we will also explore the effect of longer PEG linkers on potency and protease sensitivity of N-linked peptides and determine the basis for the difference in antiviral spectrum conferred by orientation of the lipid moiety. Future studies will address the issue of protease sensitivity, in addition to varying the location of the lipophilic moiety, by modifying the amino acid backbone so as to render it less protease sensitive.

The biophysical data shown here lead to several conclusions that can now be applied to peptide design. The lipid moiety drives the interaction between the peptides and the lipid membrane, and is a determinant for the affinity of the compounds toward the membrane. As the cholesterol moiety is conserved among the active compounds we assessed here, it may explain the similar dissociation constants obtained for the different molecules. However, we observed significant differences in the kinetic behavior of each peptide. VG-PEG24-Chol exhibits the fastest kinetics of membrane insertion, followed by VG-PEG4-Chol. The addition of cholesterol to the lipid monolayers boosts the kinetic rate constant of all peptides; only the most potent peptides interact well with membranes in the absence of cholesterol in the target membrane (data not shown). We speculate that the linear membranotropic (association/dissociation from membrane) properties associated with the effective peptides also affect biodistribution and therefore *in vivo* efficacy, as they may circulate in association with RBCs. We assessed the serum and RBCs from peptide-dosed animals, and found that peptides are present both in the plasma and on the surface of the RBCs (data not shown). Association with RBCs may be one mechanism whereby the lipid moieties enhance half-life *in vivo*. Addition of PEG linkers, while significantly enhancing potency, confers protease sensitivity, an issue that may be mitigated by measures including N-terminal conjugation. This understanding of the set of properties that correlate with the peptides’ potency permits enhancement of antiviral effect of HPIV3-derived peptides for both HPIV3 and NiV. In addition to guiding peptide design, these principles will be used to obtain the optimal balance of orientation, lipid moiety, valency, linker length, and amino acid sequence with the goal of broad spectrum activity for paramyxoviruses.

## Methods

### Cells

293T (human kidney epithelial), CV-1 and Vero E6 cells were obtain from ATCC and were grown in Dulbecco’s modified Eagle’s medium (DMEM) (Gibco) supplemented with 10% fetal bovine serum and antibiotics at 37 °C and 5% CO_2_. All cells were tested negative for mycoplasma in MycoAlert™ Mycoplasma Detection Kit (Lonza).

### Peptide synthesis

All peptides were produced by standard Fmoc-solid phase methods. The cholesterol moiety was attached to the peptide via chemoselective reaction between the thiol group of an extra cysteine residue, added C-terminally to the sequence, and a bromoacetyl derivative of cholesterol, as previously described^8^,^24^.

### Viruses

HPIV3 clinical isolate virus (CI) was obtained from the Clinical Microbiology Laboratories at New York Presbyterian Hospital and grown in human airway epithelium (HAE) at an air-liquid interface for only one passage prior to use in these experiments. Recombinant virus expressing GFP^25^ used in the cotton rat experiments was obtained from Ursula Buchholz and Peter Collins (NIAID).

Pseudotyped viruses were generated using VSV-ΔG-RFP, a recombinant VSV derived from the cDNA of VSV Indiana in which the G gene is replaced with the Ds-Red gene (RFP). Pseudotypes with NiV F and G were generated as described previously^8^. Briefly, 293T cells were transfected with plasmid encoding VSV-G or NiVF/G. Five hours post-transfection, the dishes were washed and infected (multiplicity of infection [MOI] of 0.5) with VSV-ΔG-RFP complemented with VSV-G. Supernatant fluid containing pseudotyped virus (NiV F/G or VSV-G) was collected 18 h post-infection and stored at −80 °C.

NiV isolated from the cerebrospinal fluid of a patient was received from Dr. K.B. Chua and Dr. S.K. Lam (University of Malaya, Kuala Lumpur, Malaysia). NiV was prepared by infecting Vero-E6 cells as previously described^26^, in the INSERM Jean Mérieux biosafety level 4 (BSL-4) laboratory in Lyon, France.

### ß-Gal complementation-based fusion assay

We previously adapted a fusion assay based on alpha complementation of ß-galactosidase (ß-Gal)^27^,^28^. In this assay, receptor-bearing cells expressing the omega peptide of ß-Gal are mixed with cells co-expressing envelope glycoproteins and the alpha peptide of ß-Gal, and cell fusion leads to alpha-omega complementation. Fusion is stopped by lysing the cells and, after addition of the substrate (^®^The Tropix Galacto-**Star**™ chemiluminescent reporter assay system, Applied Biosystem), fusion is quantified on a Spectramax M5 microplate reader. In the overnight fusion assay, cells expressing both alpha and omega peptides of ß-Gal were co-cultured for up to 20 h.

### Pseudotyped entry assay mimicking multicycle replication

As described previously^29^,^30^, NiVF/G glycoproteins were pseudotyped onto VSV-ΔG–RFP and the resulting pseudotyped viruses were used to infect NiV F/G-expressing cells, at an MOI of 0.125, for simulation of multicycle replication. RFP production at 24, 48, 72 and 96 h was analyzed on a microplate fluorescence reader (Spectramax M5). For detecting RFP expression levels, the wells were read by excitation at 535-nm and emission at 579-nm. For the detection of YFP expression, the wells were read by excitation at 510-nm and emission at 535-nm. For the modified multicycle replication assay, VSV G glycoprotein was pseudotyped onto VSV-ΔG–RFP and the resulting pseudotyped viruses were used to infect viral glycoprotein(s)-expressing cells for a simulation of multicycle replication. For single-cycle infection assays, the VSV-G pseudotype was used at an MOI of 0.125 to infect 293T cells transfected with control plasmid.

### Assay for triggering of F-mediated fusion using uncleaved HPIV3 F K108G: receptor retention, receptor release, and F retention by peptides

Monolayers of 293T cells were transfected to transiently express the cleavage site mutant HPIV3 F (K108G) and WT HN in the presence of neuraminidase 40 mU/ml, overnight. The next day, cells were washed and incubated with neuraminidase 40 mU/ml and cyclohexamide 1:1000 (100 μg/μL), for 1 h, at 37 °C. Then, cells were washed and incubated with 1% RBC suspensions in CO_2_ independent medium (Life Technology) at pH 7, for 30 min, at 4 °C. After rinsing to remove unbound RBCs with CO_2_ independent medium, 1 μM of peptide was added, and the plates were placed at 37 °C for 1 h. Zanamivir was added at a final concentration of 2 mM, and the plates were left at 37 °C until the complete release of RBCs in the non-treated samples. Plates were rocked, and the liquid phase was collected in V-bottomed tubes for measurement of released RBCs. The monolayers were incubated at 4 °C with 200 ml of RBC lysis solution (lysis of unfused RBCs with NH_4_Cl removes the RBCs whose membranes have not fused with HN/F-coexpressing cells). The liquid phase was collected in V-bottomed 96-well plates for the measurement of reversibly bound RBCs. The cells then were lysed in 200 μl 0.2% Triton X-100-phosphate-buffered saline (PBS) and were transferred to flat-bottomed 96-well plates for quantification of the pool of fused RBCs. The amount of RBCs in each of the three compartments described above was determined by the measurement of absorption at 405 nm.

### Lipids

POPC was purchased from Avanti Polar Lipids (Alabaster, AL, USA), while cholesterol (Chol) was from Sigma.

### Fluorescence spectroscopy measurements

VG peptides contain one tryptophan residue, intrinsically fluorescent, which makes fluorescence spectroscopy methodologies suitable tools to follow these molecules. Membrane dipole potential studies were carried out in a Varian Cary Eclipse fluorescence spectrophotometer (Mulgrave, Australia). Peptide aggregation studies were evaluated in a FLS920 series fluorescence spectrophotometer (Edinburgh Instruments, Livingston, UK). L-Tryptophan, HEPES and NaCl were from Merck (Darmstadt, Germany). The working buffer used throughout the studies was HEPES 10 mM pH 7.4 in NaCl 150 mM^16^,^31^,^32^.

Intrinsic fluorescence measurements of VG peptides and tryptophan (for the sake of comparison) were performed with an excitation wavelength of 280 nm and a starting emission wavelength of 300 to 450 nm. The excitation spectra of the same species was obtained from 220 to 300 nm, with a fixed emission wavelength of 350 nm.

ANS fluorescence emission spectra were obtained from 400 to 600 nm, with an excitation wavelength of 369 nm. Typical spectral bandwidths were 5 nm for excitation and 10 nm for emission. Excitation and emission spectra were corrected for wavelength-dependent instrumental factors^33^. Emission was also corrected for successive dilutions, scatter and simultaneous light absorptions of quencher and fluorophore^34^. All fluorescence measurements were performed at approximately 25 °C.

### Aggregation followed by ANS fluorescence

The effect of peptide concentration on VG, VG-Chol, VG-PEG4-Chol or VG-PEG24-Chol aggregation was followed by ANS fluorescence^35^,^36^, with excitation at 369 nm. The intensity of fluorescence emission was collect from 400 to 600 mn. A final ANS concentration of 12.8 *μ*M was used throughout the experiments and titrated with peptides stock solution to yield a final peptide concentration in the range of 0–8 *μ*M. 2 way-ANOVA statistical analysis was applied to distinguish the influence of PEG linkers in peptide aggregation.

### Surface pressure

Changes on the surface pressure of lipid monolayers induced by VG, VG-Chol, VG-PEG4-Chol or VG-PEG24-Chol were measured in a Langmuir-Blodgett trough NIMA ST900 (Coventry, UK), at constant temperature (25 ± 0.5 °C).

Briefly, a solution of lipids in chloroform was spread on a Teflon trough of fixed area until it reaches a surface pressure of 23 ± 1 mN/m. The lipid used in this study forms monolayers upon spreading on the air–water interface. Peptide solutions were injected in the subphase and the changes on the surface pressure were followed during the time necessary to reach a constant value. The surface pressure of an air-water interface upon injecting the largest concentration of each peptide used throughout the studies was always below 15 mN/m (data not shown). For this reason, the lowest initial surface pressure of the lipid monolayers before the addition of the peptides to the subphase was above that value. In this condition, the changes in surface pressure observed upon the injection of the peptide can be attributed to an effect of the peptide on the monolayer interfacial tension.

The dissociation constant (*K*_d_) was calculated from the adsorption Langmuir isotherm:


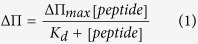


where ΔΠ are the changes of surface pressure, ΔΠ_max_ is the maximum change of pressure achieved and [peptide] is the peptide concentration.

The insertion rate constant (*k*) was calculated from the equation^37^:


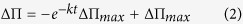


### Membrane dipole potential assessed by di-8-ANEPPS

Isolation of RBCs from human blood samples was approved by the joint ethics committee of Faculdade de Medicina da Universidade de Lisboa and Hospital de Santa Maria. These samples were obtained from healthy volunteers at the Instituto Português do Sangue (Lisbon, Portugal). All patients provided written informed consent to be enrolled in the study. In addition, all methods were performed in accordance with the relevant guidelines and regulations.

RBCs were labeled with the membrane dipole potential sensitive probe di-8-ANEPPS (Invitrogen, Carlsbad, CA, USA). Briefly, blood samples were centrifuged at 1200 × *g* for 10 min, plasma and buffy-coat were removed, and remaining RBCs were washed twice with HEPES buffer. RBCs at 1% hematocrit were incubated in buffer supplemented with 0.05% (m/v) Pluronic F-127 (Sigma) and di-8-ANEPPS 10 μM during 1 h^17^,^38^. The unbound probe in the labeled cells was washed with Pluronic-free buffer on two centrifugation cycles. VG, VG-cholesterol, VG-PEG4-Chol and VG-PEG24-Chol were incubated with erythrocytes at 0.02% hematocrit, during 1 h, with gentle agitation, before fluorescence measurements.

Excitation spectra and the ratio of intensities at the excitation wavelengths 455 and 525 nm (*R* = *I*_455_/*I*_525_) were obtained with emission set at 670 nm, in order to avoid membrane fluidity-related artifacts^39^. Excitation and emission slits for these measurements were set to 5 and 10 nm, respectively. The variation of R with the peptide concentration was analyzed by a single binding site model^40^,^41^:


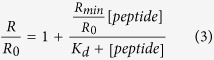


with *R* values normalized for *R*_0_, the value in the absence of peptide. *R*_min_ defines the asymptotic minimum value of *R* and *K*_*d*_ is the apparent dissociation constant.

### Antiviral activity against live HPIV3

Peptide activity against HPIV3 was determined by plaque reduction assays in infected cell monolayers^18^. IC50 of inhibition was calculated using Graph Pad Prism 5 software. Data were expressed as mean ± standard deviation (s.d.) (*n* = 3 separated experiments).

### Antiviral activity against live Nipah virus

In the plaque reduction assay, serial dilutions of peptides in Dulbecco’s modified Eagle’s medium (DMEM) (Invitrogen) were added to reporter cell monolayers of Vero E6 cells (in 6 well-plates, 106 cells/well) for 1 h at 37 °C, 5% CO_2_. Then, 200 pfu of NiV were added to the supernatant fluid of the Vero cell culture and incubated for 1 h at 37 °C. Supernatant fluids were then replaced by 2 ml of 1.6% of carboxymethylcellulose in DMEM containing 3% of FCS. Plates were incubated for 4 days at 37 °C, 5% CO_2_. Plates were developed using a central plaque assay, as previously described^26^. IC50 of inhibition were calculated using Graph Pad Prism 5 software. Data were expressed as mean ± s.d. (*n* = 6 separated experiments).

### Cell viability assay

Vero cells were seeded in 96-well plates at a density of 5 × 10^3^ cells/well, in DMEM (Gibco) supplemented with 10% FCS and 2 mM L-glutamine and allowed to form confluent monolayers overnight at 37 °C in a humidified CO_2_ (5%) atmosphere. Cell cultures were then incubated at 37 °C in the absence or presence of serial dilutions of peptides. Cell viability was determined after 24 hrs at 37 °C by the MTT method (Vybrant^®^ MTT Cell Proliferation Assay Kit V-13154).

### HAE cultures

The EpiAirway AIR-100 system (MatTek Corporation) consists of normal human-derived tracheo/bronchial epithelial cells that have been cultured to form a pseudostratified, highly differentiated mucociliary epithelium closely resembling that of epithelial tissue *in vivo*. Upon receipt from the manufacturer, HAE cultures were transferred to 6-well plates (containing 0.9 ml medium per well) with the apical surface remaining exposed to air and incubated at 37 °C in 5% CO_2_.

### Peptide efficacy assessment in HAE cultures

HAE cultures were infected by applying 100 μl of EpiAirway medium containing 4,000 PFU of HPIV3 with or without peptide to the apical surface for 90 min at 37 °C. The medium containing the inoculum was removed, and cultures were placed at 37 °C and fed each day with 0.9 ml medium via the basolateral surface. Viruses were harvested by adding 200 μl medium per well to the HAE cultures’ apical surface and allowed to equilibrate for 30 min at 37 °C. The suspension was then collected, and viral titers were determined as previously described^18^. Viral collection was performed sequentially with the same wells of cells on each day post infection.

### Protease sensitivity of HPIV3 derived peptides

For the trypsin digestion, 1 μg of each peptide was treated with 0.025 μg of trypsin in 10 μl of 1X PBS. Peptide solutions were then incubated at 0 °C or 37 °C for 30 minutes. Following incubation, 10 μl of Laemmli’s SDS reducing buffer was added to each solution. Samples were boiled for 10 minutes at 99 °C then run on a 10–20% Tricine gel at 120 V. The gel was allowed to fix overnight in a 0.0125% glutaraldehyde in PBS. Gels were stained using a Pierce^®^ Silver Stain kit (Cat# 24600).

### Biodistribution analysis

#### Animal experiment

6–8 week old female Syrian golden hamsters, 3 animals per group, were intraperitoneally injected with peptides (2 mg/kg) in 400 μL of water for injection (Aguettant). At each time point, blood was collected in EDTA vacutainer tubes from all animals of the corresponding group, tubes were centrifuged for 10 min at 2000 RPM, and plasma was transferred into new tubes and conserved at −20 °C for use in ELISA. At 24 h, organs from each animal were collected and separated in two: one half was conserved at −80 °C for ELISA, the other half frozen in cold isopentane on dry ice and placed at −80 °C for cryosectioning. Animals were bred at the animal facility in UMDNJ Newark and the protocols were approved by the Institutional Animal Care and Use Committee (IACUC Approval for Protocol Amendment #P129A1). All the experiments were performed in accordance with the relevant guidelines and regulations.

#### Preparation of organs for ELISA

Each frozen organ was weighed and mixed into 1X PBS (1:1, w/v) using an ultra turrax homogenizer. Samples were then treated with acetonitrile/TFA 1% (1:4, v/v) for 1 h on a wheel at 4 °C and then centrifuged for 10 min at 8000 rpm. Supernatant fluids were collected for ELISA.

#### ELISA

96 well plates Maxisorp (Nunc) were coated overnight with purified goat anti-HPIV3 F HRC antibodies in carbonate/bicarbonate buffer pH = 7.4 (20 μg/ml). Plates were then washed twice in 1X PBS and blocked in 3% BSA/1X PBS for 30 min. The blocking buffer was replaced by 2 dilutions of each sample in 3% BSA/1X PBS in duplicate and incubated for 1.5 h at room temperature (RT). Wells were washed 3 times in 1X PBS, and developed with purified rabbit anti-HPIV3 F HRC antibodies conjugated to peroxidase (1/1500) in 3% BSA/1X PBS for 1 h at RT, followed by 5 washes and incubation with the Sigmafast OPD substrate system (Sigma-Aldrich, France), and stopped with sulfuric acid (12%). The optical density was read at 492 nm.

#### Immunofluorescence

The cryo-sections were dried for 30 min and fixed in a 4% formalin solution. After multiple washes in PBS, saturation was performed using PBS/4% FBS (30 min at RT) before incubation with specific rabbit anti-HPIV3 F HRC antibody (overnight at 4 °C) in PBS/4% FBS. After multiple washes, tissue sections were incubated with the secondary goat anti-rabbit antibody conjugated with Alexa 488, in PBS/4% FBS (2 h, RT). The nuclei were counterstained with DAPI. After multiple washes in PBS, mounting was performed using Fluoroprep (BioMérieux). Brain sections were analyzed using an Axioplan 2 imaging microscope (Zeiss).

### Golden hamster infection

Eight-week-old female Syrian golden hamsters (*Mesocricetus auratus*, Janvier, France), randomly distributed into cages by animal care takers, were anesthetized and infected intraperitoneally (i.p.) with 0.4 mL of NiV (100LD_50_) in BSL-4 conditions. Groups of 6 animals were treated daily with i.p. injections of peptide (2 mg/kg) for 10 days, starting from the day of infection, or received the vehicle in the untreated group without formal randomization. The animals were followed daily for 3 weeks in a blinded fashion.

### Cotton rat infection

Inbred cotton rats were obtained from Harlan (Indianapolis, IN). Female animals, 7 weeks of age, were randomly distributed into cages by animal care takers. After acclimatization of one week, cages of cotton rats were grouped without formal randomization into treatment groups and infected with 10^6^ pfu intranasally (i.n.), as previously described^42^,^43^. Groups of 4 animals were infected with the indicated virus and treated s.q. with 4 mg/kg/day of peptide, 2 doses per day, for day −1 to day 2 after infection. Group size was based on power analysis to determine a difference in treatment of at least one log10 in viral titer. Viral titers were determined 3 days post infection as previously described^42^. No samples were excluded from the analysis. No significant variance between groups was detected and samples were analyzed in a not blinded fashion. Animal work was approved by the Institutional Animal Care and Use Committee by the Ohio State University, and were performed in accordance with the relevant guidelines and regulations.

### Statistical Analysis

All data were normally distributed with equal variance. Data are described as mean ± s.d. unless otherwise stated. For the peptide aggregation experiments, a 2-way ANOVA analysis was performed to distinguish different peptides and concentrations. Bonferroni post-tests were performed to compare replicate means. The statistical analysis performed on *in vivo* experiments for comparison of HPIV3 viral titers in cotton rats lungs were based on the non-parametric Mann–Whitney *U*-test, and Mantel-Cox analysis for comparison of survival curves in NiV infected golden hamsters. All the data and statistical tests were performed in Prism 5 (GraphPad Software, La Jolla, CA).

## Additional Information

**How to cite this article**: Mathieu, C. *et al*. Broad spectrum antiviral activity for paramyxoviruses is modulated by biophysical properties of fusion inhibitory peptides. *Sci. Rep.* **7**, 43610; doi: 10.1038/srep43610 (2017).

**Publisher's note:** Springer Nature remains neutral with regard to jurisdictional claims in published maps and institutional affiliations.

## Figures and Tables

**Table 1:** Cytotoxicity, *in vitro* activity, and specificity of fusion inhibitory peptides.

Peptide	*Cytotoxicity in monolayer culture*	Efficacy in plaque reduction assay vs. HPIV3	
	CC_50_ (nM)^a^	IC_90_ (nM)^b^	IC_50_ (nM)^b^
VG	>10,000	280 ± 247	1
VG-Chol	10,000	0.7 ± 0.26	0.015 ± 0.07
VG-PEG4-Chol	4,500	0.7 ± 0.007	0.03 ± 0.04
VG-PEG24-Chol	1,300	0.1 ± 0.0003	0.007 ± 0.007
Chol-VG	9,000	1.7 ± 0.42	0.06 ± 0.035
Chol-PEG4-VG	>10,000	0.1 ± 0.0001	<0.0007
HRC_FIV_ PEG 24 Chol	9,000	>9,000	>9,000
HRC_MV_ PEG 24 Chol	2,000	>2,000	>2,000
HA_FLU_ PEG 24 Chol	3,000	>3,500	>3,500

^a^Compound concentration required to reduce the viability of mock-infected Vero cells by 50%, as determined by MTT.

^b^Compound concentration required to achieve 50% or 90% plaque reduction for HPIV3.
